# Comparison between transoral laser surgery and radiotherapy in the treatment of early glottic cancer: A systematic review and meta-analysis

**DOI:** 10.1038/s41598-018-30218-x

**Published:** 2018-08-09

**Authors:** André Vicente Guimarães, Rogério Aparecido Dedivitis, Leandro Luongo Matos, Felipe Toyama Aires, Claudio Roberto Cernea

**Affiliations:** 1 Department of Head and Neck Surgery, Hospital das Clínicas, São Paulo School of Medicine, São Paulo, Brazil; 2 ENT and Head Neck Discipline, University Metropolitana de Santos, UNIMES, Santos, Brazil; 3 Department of Head and Neck Surgery, São Paulo School of Medicine, University of São Paulo, São Paulo, Brazil

## Abstract

A therapeutic decision in the treatment of Tis/T1a glottic carcinoma with radiotherapy (RT) or transoral laser surgery (TOS) is still an open issue. Oncologic outcome and voice quality may support the choice for the latter To conduct a systematic review and meta-analysis to compare oncologic and functional outcomes of TOS and RT as treatment options for Tis/T1a glottic cancer. Literature research on online databases was carried out. Potentially eligible articles were reviewed. Relevant articles were selected and evaluated. There was statistical significance favoring patients initially treated with TOS when it comes to overall survival, disease-specific survival and larynx preservation. No difference in local control was found. TMF, *Jitter* and *Shimmmer* measurements presented statistically significant results in favor of RT. Self-assessment of voice quality (VHI) and f0 showed no statistically significant differences. Maximum Phonation Time (MPT) had a better response to RT. There is a trend in favor of RT. Tis/T1a glottic cancer patients submitted to TOS had significant overall and disease specific survival and had fewer risks of having a total laryngectomy, when compared to the radiotherapy group. The self-assessment of voice quality and f0 did not show any difference; however, *Jitter*, *Shimmer* and MPT measurements favored radiotherapy.

## Introduction

The aim of the treatment of early glottic carcinoma (T1-T2) is to achieve local control of the disease, preserving the organ as well as its functions (breathing, protection of the airways and phonation). It can be treated by transoral laser microsurgery (TOS) of the larynx or by cold instrument, open partial laryngectomy or radiotherapy (RT). All these modalities present good oncologic results^1^.

Early vocal fold cancer rarely presents metastasis due to the scarce lymphatic sub-mucosa network^2^. Therefore, only local treatment in the carcinoma in stage T1 of the glottis (via endolaryngeal endoscopic or via exclusive radiotherapy) serves oncologic precepts.

Each therapeutic modality presents advantages and disadvantages. Endoscopic resection allows targeted resection and tissue sparing, but requires specific training and surgical equipment. Some patient’s anatomy does not allow good anatomical exposure of the larynx, resulting in either an aborted procedure or inadequate oncologic resection. Radiotherapy on the other hand is more widely available, can be delivered using standard external beam radiation sources, and has a low risk of having to convert to alternate therapy. The disadvantages of radiotherapy, however, are extensive including local temporary or persistent edema, glottis stenosis, xerostomia, and hypothyroidism. Throughout the literature both methods have presented good oncologic and functional results; however, the choice taken depends on the experience and preference of the group in charge of the patient, as there are no clear-cut advantages of one over the other^1,3^.

The objective is to perform a systematic review and meta-analysis in order to compare oncologic and functional outcomes of TOS and RT as treatment options for Tis/T1a glottic cancer.

## Methods

### Selection criteria

Epidemiological studies comparing endoscopic resection to radiotherapy in patients with early-stage glottic carcinoma were evaluated. Studies were selected from the reading of respective titles and abstracts. When it was not possible to identify if the study would be included or not, the full text was requested for a more detailed analysis.

Studies including patients with a confirmed diagnosis of glottic squamous cell carcinoma (SCC) classified according to the TNM-UICC (7^th^ edition), such as T1aN0M0 were considered. Studies that evaluated patients previously treated with surgery and/or radiotherapy in the head and neck were excluded.

The intervention group was considered as the patients submitted to endoscopic resection. A priori, there was no difference regarding technique and/or material used for the performance of the surgery. The control group was considered as the patients that were submitted to radiotherapy. No distinction was made in relation to the characteristics of the technology and/or dosage given. The evaluated outcomes were overall and specific mortality (survival), local control (recurrence) and vocal quality. Vocal quality was assessed by means of objective variables (fundamental frequency measurements, maximum phonation time, *Jitter, Shimmer* and harmonic-noise ratio - HNR) regardless of the method used and the subjective variables (*VHI* – *Voice Handicap Index*).

### Identification and selection of the studies

A strategy of literature survey was employed in order to perform the systematic review of the available evidence. This included research on the following data basis: Medline (through PubMed) and Lilacs up to October, 2016.

The survey strategy *(laryngeal neoplasms OR early glottic cancer OR early glottic carcinoma OR early vocal cord cancer) AND (endoscopic resection OR surgery OR laser surgery OR gas lasers OR laser therapy OR cordectomy) AND (radiotherapy) AND (epidemiologic studies OR comparative studies OR randomized controlled trial)* was employed.

Furthermore, references from the articles selected were also evaluated in order to select studies not covered in the electronic survey.

### Statistical analysis

The measurements of effectiveness or damage expressed through absolute values were analyzed by means of the absolute risk difference, under the 95% confidence interval (CI). The values necessary to treat and the values necessary to cause damage were calculated respectively for all the statistically significant results. The continuous data were analyzed regarding their means and SDs. The difference between the weighted mean of the groups was used for the analysis. Inconsistencies among the clinical trials were estimated by using the heterogeneity Chi-square test (Chi^2^) and quantified by the Test I^2^. Values above 50% were considered to be of high heterogeneity.

## Results

### Results from electronic searches

**Figure 1:**
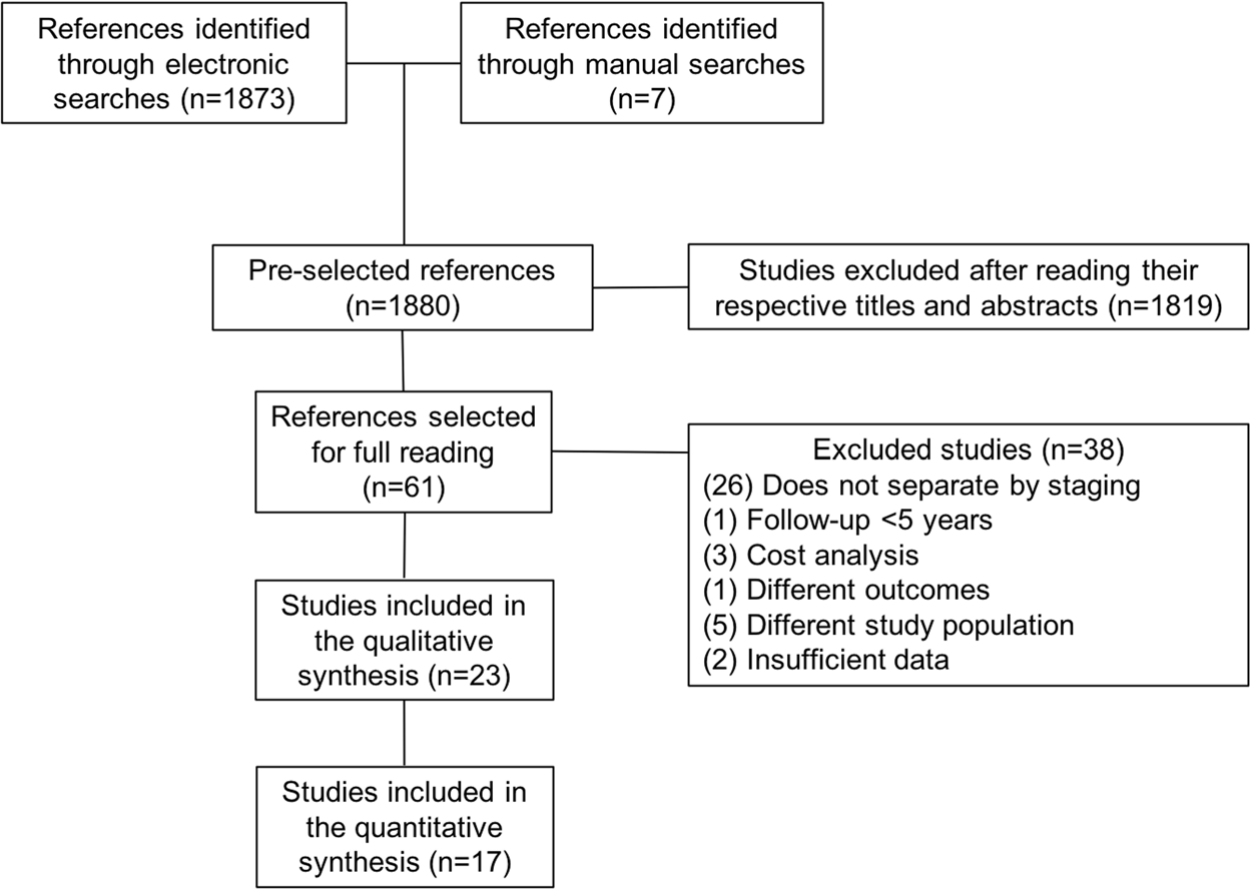
Selection flow of articles for the systematic revision.

1.873 studies were identified by electronic search and seven by manual search. 1.819 studies which clearly did not meet the inclusion criteria by the reading of their titles and abstracts were excluded. 61 were considered potentially eligible, from which 23 were included (Fig. 1). Just one controlled and randomized clinical trial was recovered, but it was not selected for revision as it evaluated the oncologic outcomes with only a 2-year follow-up and vocal quality was evaluated by using the GRBAS scale^4^.

Of the studies included, 5 are prospective cohort^5–9^ and the other 17 are retrospective^10–26^. All of them presented a well-defined inclusion criteria, with patients with early glottic carcinoma (Tis-T1a) with no previous history of malignity or manipulation of the vocal folds. The allocation of patients for this type of treatment was extremely heterogeneous (patient’s decision, medical decision, study period, institutional protocol and findings in laryngostroboscopy). In half of these studies this criterion was uncertain.

### Overall and specific mortality

**Figure 2:**
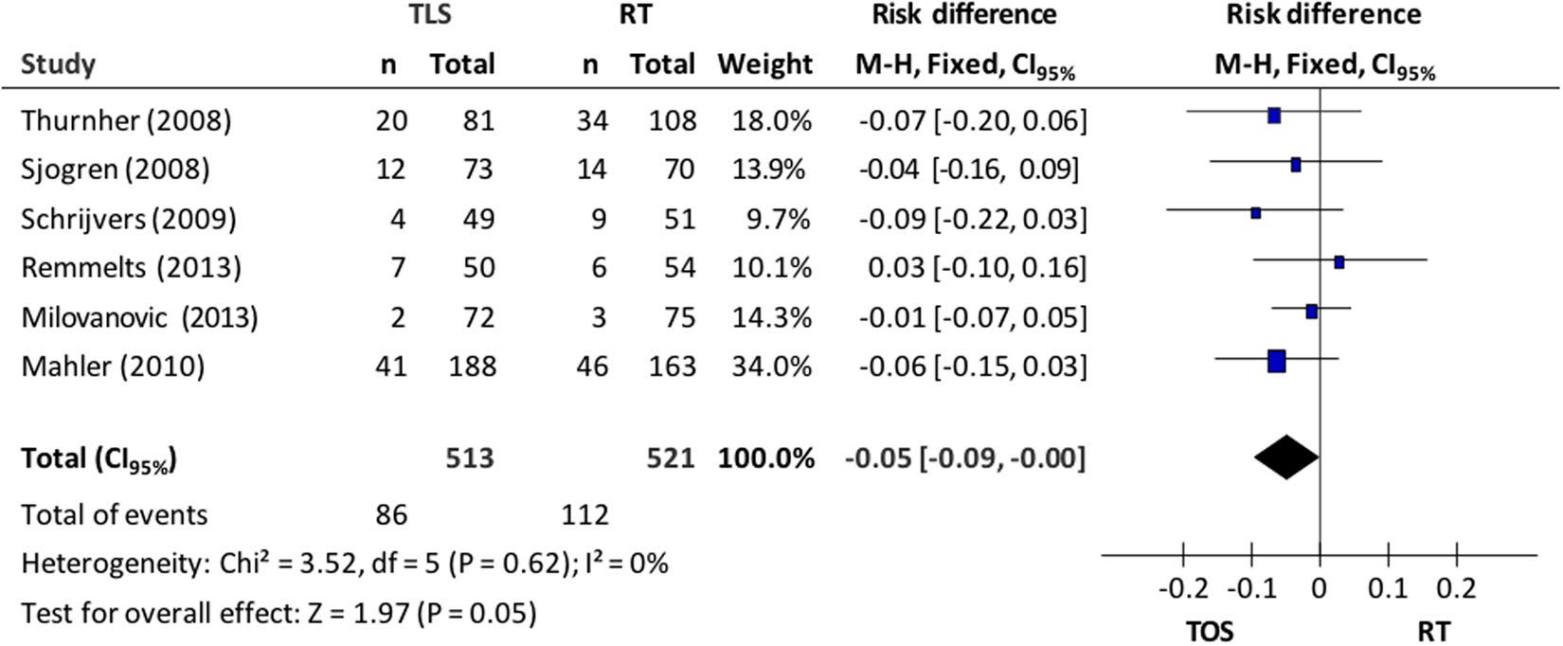
*Forest plot* chart comparing microsurgery to radiotherapy in relation to overall survival.

Overall mortality (6 studies) was 19.1%: 16.8% in the endoscopic resection group and 21.5% in the RT group. A fixed-effects model was used due to the lack of heterogeneity between the studies (Chi^2^ = 3.52; p = 0.62; I^2^ = 0%). Meta-analysis of the data showed that patients submitted to microsurgery presented greater overall survival when compared to the RT group (CI_95%_, 0.00 to 0.09; p = 0.05; NNT = 20;)–Fig. 2.

**Figure 3:**
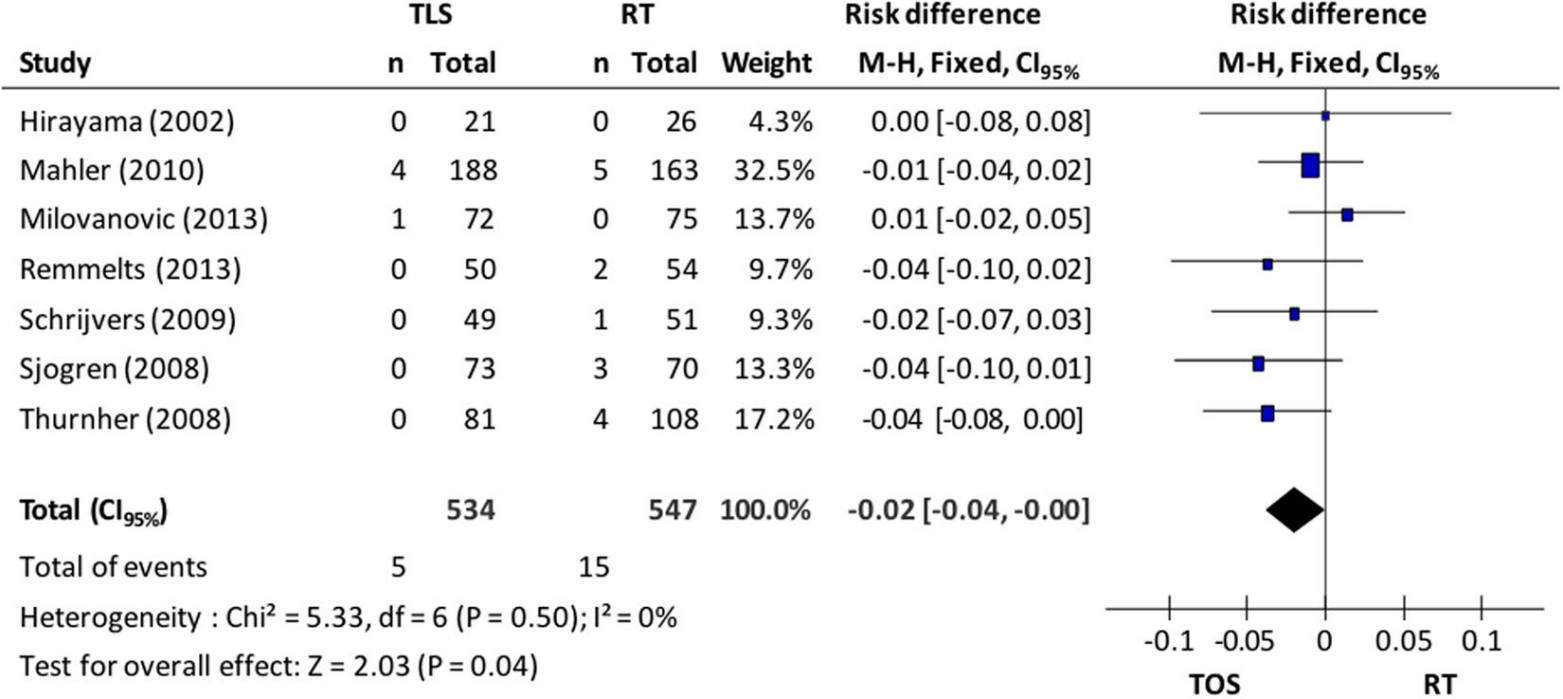
Chart shows *Forest plot* comparing microsurgery to radiotherapy in relation to specific mortality.

In relation to the specific mortality from the disease (7 studies), the TOS group presented 5 cases (0.9%) and the RT group, 15 cases (2.7%). Using the fixed-effects model (I^2^ = 0%), the meta-analysis resulted in a discreet reduction in death risk from the disease (ARR = 2%) in the group of patients submitted to microsurgery (CI_95%_, 0.00 to 0.04, p = 0.04; NNT = 50)–Fig. 3.

### Local control

**Figure 4:**
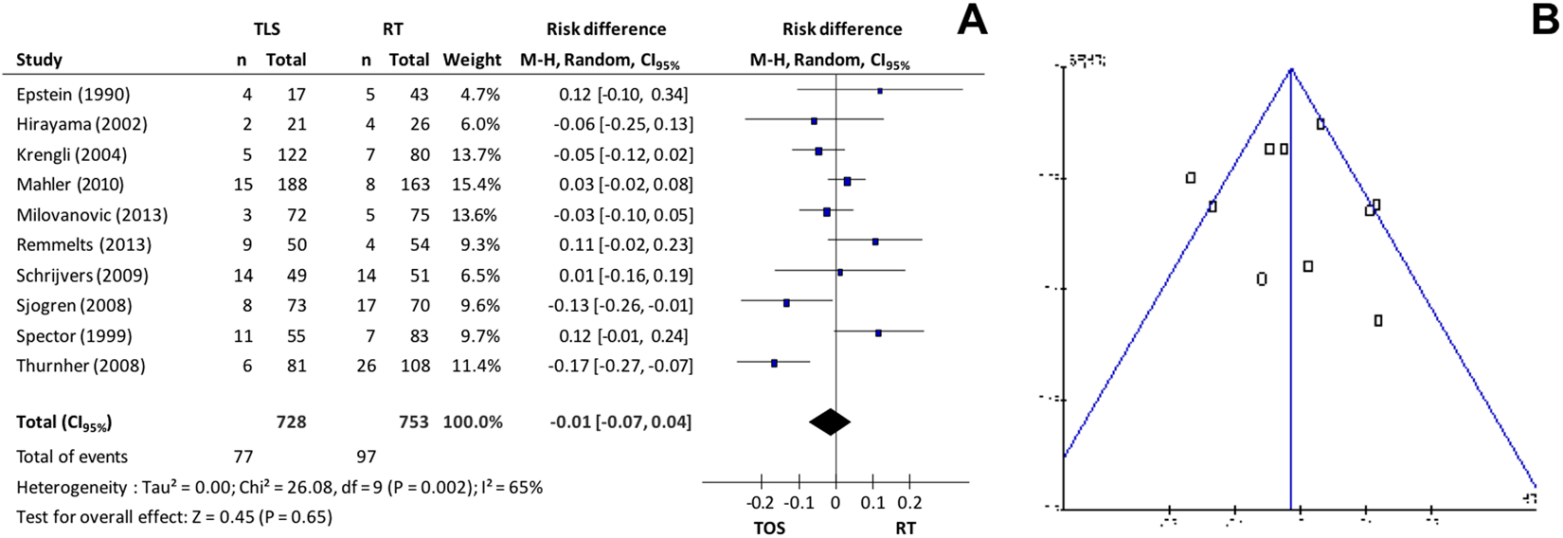
(**A**) *Forest plot* chart comparing microsurgery to radiotherapy in relation to local control and (**B**) funnel plot to identify studies with high heterogeneity in the evaluation of local control.

Local control of the disease (recurrence) was evaluated in ten studies, totaling 1.481 patients: 728 in the microsurgery group and 753 in the radiotherapy group. A random effects model was used for the analysis of recurrence incidence. Data synthesis of the primary studies showed that there is no significant difference in the local control of the disease between groups (10.6% in TOS group vs 12.9% in RT group; CI_95%_ −0.07 to 0. 04; p = 0.65; I^2^ = 65%) observed in Fig. 4A. Excluding studies responsible for high heterogeneity^10,11^, the difference between groups remained non-significant (10.9% vs 9.4%, CI_95%_ −0.01 to 0.06; p = 0.16; I^2^ = 36%) according to Fig. 4B.

## Larynx Preservation

**Figure 5:**
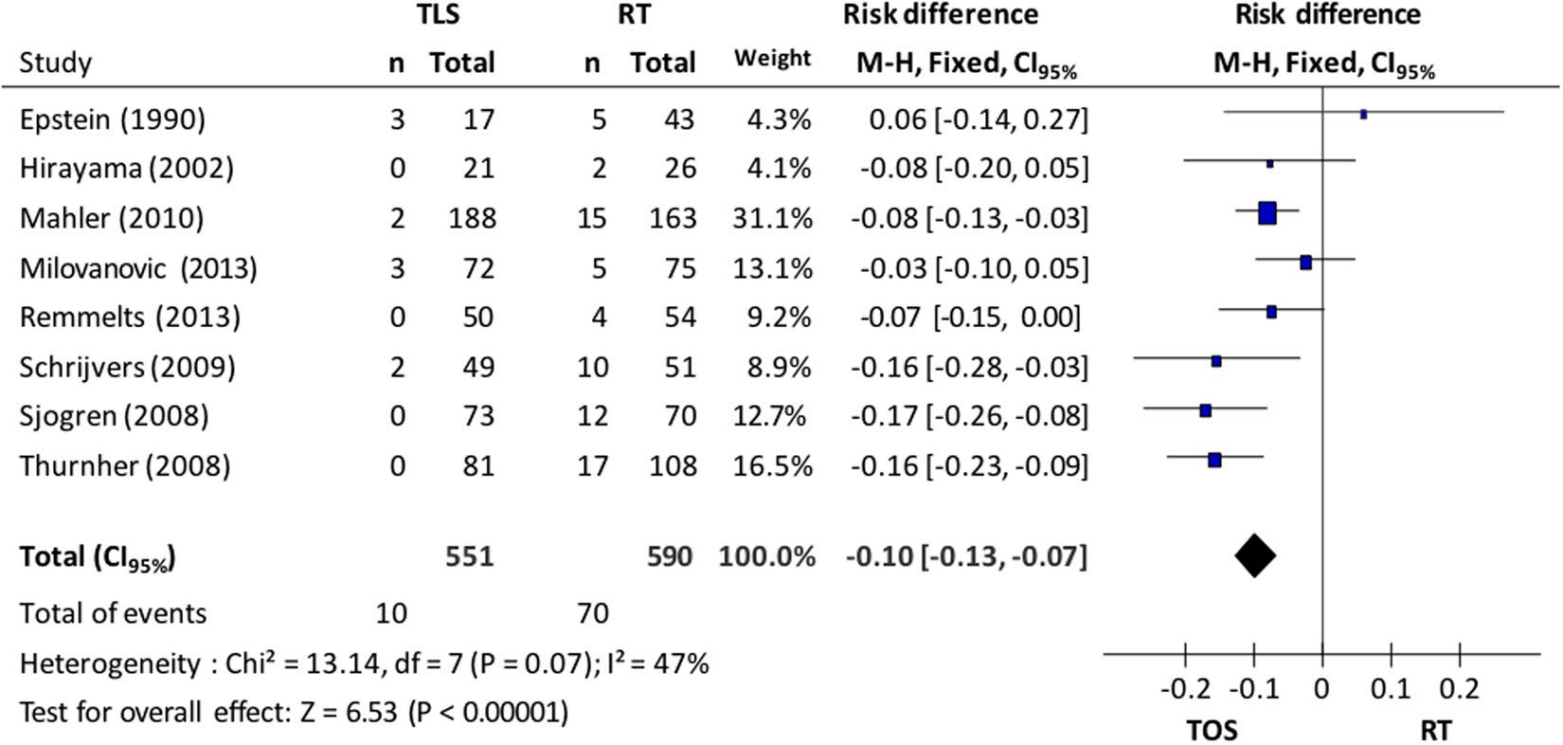
*Forest plot* chart comparing microsurgery to radiotherapy in relation to larynx preservation.

In relation to larynx preservation, 8 studies supplied data for the evaluation: 551 patients from the microsurgery group and 590 from the radiotherapy group. A fixed-effect model was used due to the heterogeneity (I^2^ = 47%). The incidence of total laryngectomy in the microsurgery group was 1.8% while in the radiotherapy group it was 11.8%; this difference was statistically significant (CI_95%_ 0.07 to 0.13; p < 0.001; I^2^ = 47%; NNT = 10), seen in Fig. 5.

### Vocal Quality

Acoustic analysis data referring to vocal quality evaluation are expressed in Tables 1 and 2. The random effects model was used for all the vocal quality analyses.

**Table 1:** Data referring to objective acoustic analyses.

Study	Transoral surgery					Radiotherapy				
	n	MPT	FO	Jitter	Shimmer	n	MPT	FO	Jitter	Shimmer
Cragle *et al*.^18^	11	16	—	0.32	13.14	20	17.5	—	0.44	16.46
McGuirt *et al*.^19^	11	15.8	157	0.74	—	13	19.3	136	0.84	—
Sjogren *et al*.^11^	18	16.2	156	0.45	4.36	15	14.5	145	1.0	5.20
Jotic A *et al*.^7^	19	—	165.5	0.68	6.82	15	—	161.4	0.68	6.9
Tamura *et al*.^21^	14	14.3 ± 6.5	169.1 ± 37.8	1.1 ± 0.8	3.8 ± 1.6	6	18.1 ± 4.4	160 ± 43.2	0.9 ± 0.6	2.8 ± 1.8
Wedman *et al*.^20^	15	—	144 ± 20	8.7 ± 2.4	1.3 ± 0.3	9	—	137 ± 12	8.4 ± 1.7	1.5 ± 0.3
Nunes Batalla *et al*.^22^	19	11.8 ± 5.3	173.4 ± 47.4	0.4 ± 0.2	5.1 ± 4.7	18	8.6 ± 3.2	199.0 ± 51.5	0.7 ± 0.9	4.1 ± 4.0
Van Gogh *et al*.^6^	55	—	141 ± 33	0.5 ± 0.5	5.3 ± 3.2	36	—	124 ± 29	0.6 ± 0.6	5.8 ± 3.8
Milovanovic *et al*.^8^	72	15.3 ± 2.1	162.4 ± 14.7	1.1 ± 0.1	3.7 ± 0.3	75	17.6 ± 2.1	159.7 ± 14.1	0.9 ± 0.1	2.8 ± 0.6
Kono *et al*.^9^	37	14.5 ± 4.2	180.6 ± 16.6	4.1 ± 0.6	7.4 ± 1.1	27	18.1 ± 4.7	167.2 ± 11.1	1.6 ± 0.3	3.4 ± 0.4

**Table 2:** Data referring to subjective acoustic analysis (*VHI*).

Study	Transoral surgery		Radiotherapy	
	n	mean ± SD	n	mean ± SD
Loughran *et al*.^23^	15	25,4 ± 24,7	18	22,2 ± 24,6
Sjogren*et al*.^11^	18	17,6 ± 8,0	15	19,2 ± 11,0
Peeters *et al*.^24^	52	18	40	12
Laoufi *et al*.^25^	43	13	30	29
Goor *et al*.^26^	42	10,6	23	17,1
Kono *et al*.^9^	37	12,6 ± 3,2	27	29,3 ± 4,9

**Figure 6:**
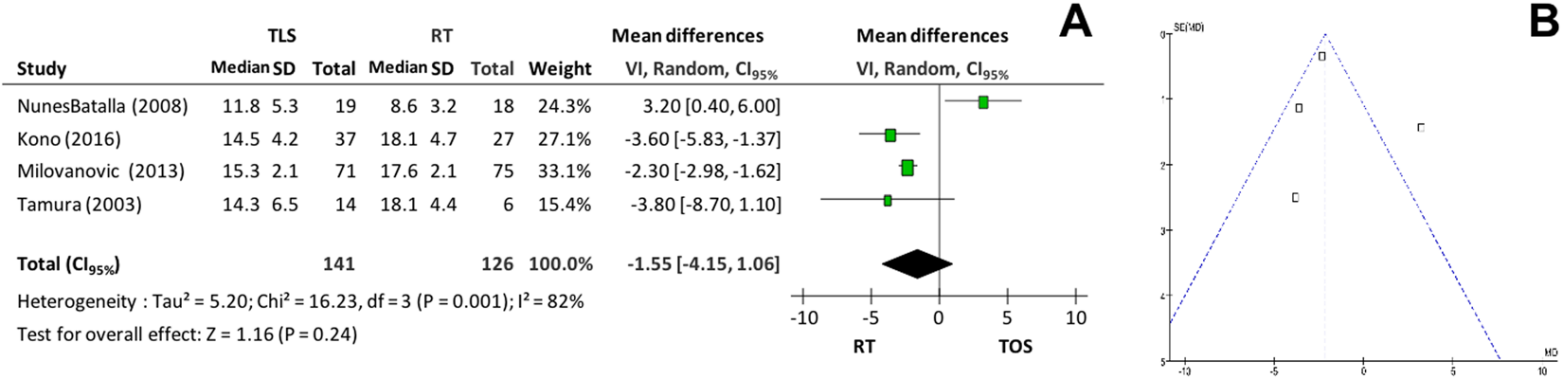
(**A**) *Forest plot* chart comparing microsurgery to radiotherapy in relation to maximum phonation time and (**B**) Funnel plot identifying a paper with high heterogeneity in the evaluation of maximum phonation time.

Regarding maximum phonation time (MPT), the microsurgery group presented variation between 11.8–16.2 seconds while the radiotherapy group varied between 8.6–19.3 seconds. There was no significant difference between the groups (p = 0.24 and I^2^ = 82%), according to Fig. 6A. However, when the study that generated high heterogeneity^22^ was excluded (Fig. 6B), the RT group presented a better result (p < 0.001 and I^2^ = 0%).

**Figure 7:**
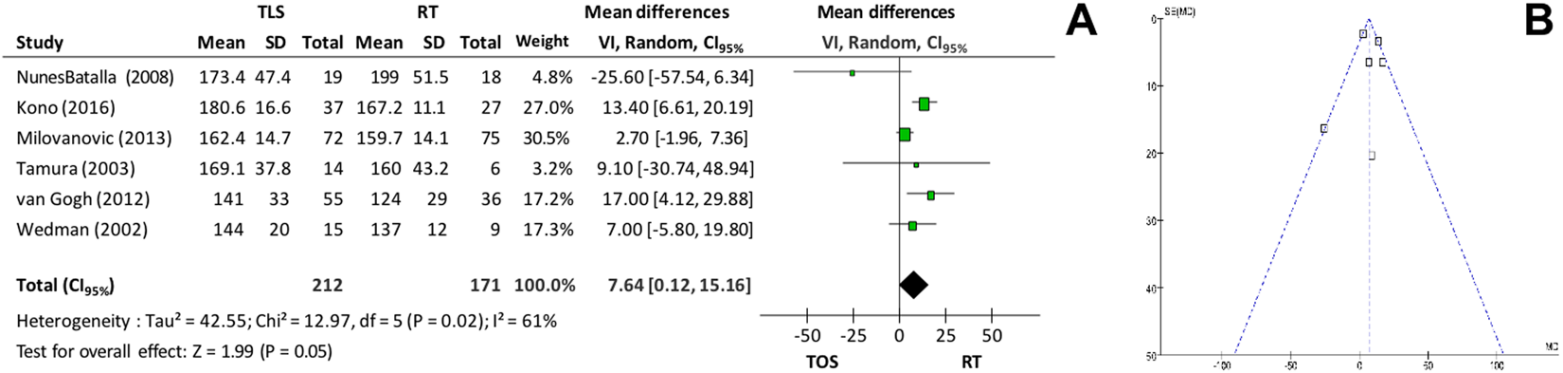
(**A**) *Forest plot* chart comparing microsurgery to radiotherapy in relation to fundamental frequency (f0) and (**B**) Funnel plot demonstrating that there was no heterogeneity in the evaluation of the fundamental frequency.

Fundamental frequency (f0) was evaluated in 9 primary studies and varied between 141–180.6 Hz in the microsurgery group and 124–199 Hz in the radiotherapy group. Meta-analysis included 6 studies and showed no significant difference between the groups (p = 0.05 and I^2^ = 61%). When the analysis was performed again, correcting high heterogeneity by removing some studies^6,9,22^, the difference between the groups remained not significant (mean difference = 3.27, CI_95%_ −1.08 to 7.63; p = 0.14; I^2^ = 0%)–Fig. 7A,B.

**Figure 8:**
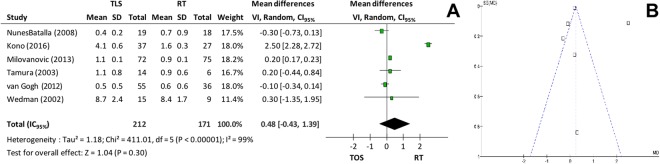
(**A**) *Forest plot* comparing microsurgery to radiotherapy in relation to *Jitte*r and (**B**) Funnel plot chart showing paper with *Jitter* heterogeneity.

**Figure 9:**
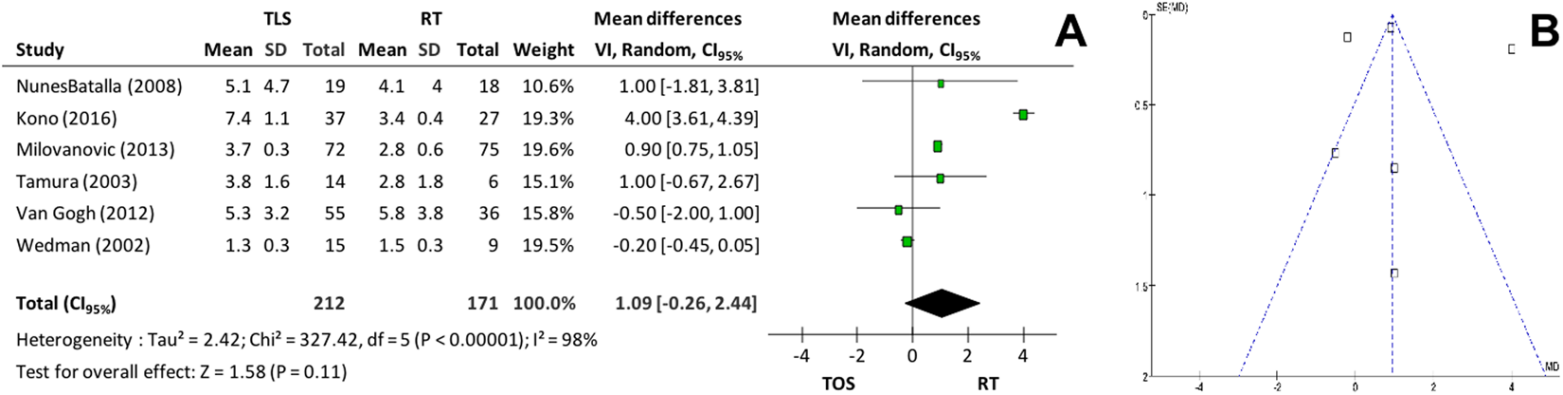
(**A**) Forest plot chart comparing microsurgery to radiotherapy in relation to *Shimmer* and (**B**) Funnel plot chart identifying 2 papers with high heterogeneity in *Shimmer*’s evaluation.

In relation to fundamental frequency perturbation (*Jitter*, Fig. 8A,B) and amplitude perturbation (*Shimmer*, Fig. 9A,B), 6 studies supplied the mean and the standard-deviations and were included in the meta-analysis. There was no significant difference for either outcome (p = 0.30 and p = 0.11, respectively). Correcting the high heterogeneities of both outcomes, lower numbers for *Jitter* (p < 0.001 and I^2^ = 0%)^6,9,22^ and for *Shimmer* (p < 0.001 and I^2^ = 10%)^9,20^ were verified.

**Figure 10:**
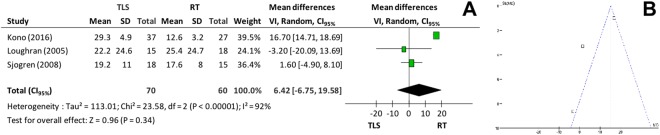
(**A**) *Forest plot chart* comparing microsurgery to radiotherapy in relation to VHI and (**B**) Funnel plot identifying 2 papers with high heterogeneity between the arms radiotherapy versus microsurgery in the *VHI* evaluation.

Subjective analysis of vocal quality was performed by using *Voice Handicap Index (VHI)* in 5 studies. In the microsurgery group, means varied between 12.6–25.4, and in the RT group, between 12–29.3. Only 3 primary studies supplied the means and standard-deviations and were included in the global analysis. Meta-analysis showed that there was no significant difference between the groups (mean difference = 6.42, CI_95%_ −6.75 to 19.58; p = 0.34; I^2^ = 92%)–Fig. 10A,B.

## Discussion

Although larynx cancer has been high prevalence, initial stage is less incident. Thus, there is a lack of prospective randomized papers with a well-designed method of patient treatment selection. The most observed bias was therapeutic selection.

For T1a glottic carcinoma there are advantages in the use of laser microsurgery as the treatment is provided in one single procedure, allows for the histopathological study of margins, preserves the contralateral vocal fold, and local recurrence can be prematurely diagnosed, permitting a punctual surgical reapproach. The disadvantages of the surgery are: removal of extra tissue with oncologic margins, thus favoring irregular glottic coaptation; the need to evaluate surgical anesthetic risk (average age around 63 years implies the existence of comorbities); the need for specialized surgery material, and inadequate exposure of the endolarynx. The advantage of radiotherapy is treating patients that do not have a good exposure of the endolarynx in the surgical field; radiotherapy also preserves more tissue, maintaining a good glottic coaptation. The disadvantage of radiotherapy is that it cannot be reused and, in recurrences, salvage is in the performance of a partially or totally open laryngectomy. Besides this, the entire larynx is irradiated, with actinic edema remaining.

Tumors in T1 stadium are still today very often submitted to radiotherapy, either because of patient’s decision, clinical contraindications for the surgery, imagining a better quality of voice, by the service protocol or medical preference.

Although overall mortality and local control have not presented statistical differences for the arms, specific mortality resulted in a discreet advantage for the microsurgery (p = 0.004). The detection of local recurrence is earlier in cases involving surgery and reapproach allows for local control as much as radiotherapy does. This can be supported by the high rates of larynx preservation in these patients (p < 0.00001). Therefore, it can be noticed that the total salvage laryngectomy is more used after radiotherapy failure.

## Conclusion

Patients with early glottic SCC Tis and T1a submitted to endoscopic resection present greater specific survival and lower risk for total laryngectomy. On the other hand, perceptive vocal quality (maximum phonation time) is superior and there is a tendency for the f0 to be closer to normal after exclusive radiotherapeutic treatment.
